# High Q-Factor, High Contrast, and Multi-Band Optical Sensor Based on Plasmonic Square Bracket Dimer Metasurface

**DOI:** 10.3390/nano14050421

**Published:** 2024-02-25

**Authors:** Bin Ni, Guanghu Chu, Zheyang Xu, Lianping Hou, Xuefeng Liu, Jichuan Xiong

**Affiliations:** 1 School of Electronic and Optical Engineering, Nanjing University of Science and Technology, Nanjing 210094, China; isweet0521@foxmail.com (G.C.); 122104222672@njust.edu.cn (Z.X.); liuxf_1956@sina.com (X.L.); jichuan.xiong@njust.edu.cn (J.X.); 2 James Watt School of Engineering, University of Glasgow, Glasgow G12 8QQ, UK; lianping.hou@glasgow.ac.uk

**Keywords:** multi-band optical sensor, surface lattice resonance, high Q-factor, high contrast

## Abstract

A high-performance resonant metasurface is rather promising for diverse application areas such as optical sensing and filtering. Herein, a metal–insulator–metal (MIM) optical sensor with merits of a high quality-factor (Q-factor), multiple operating bands, and high spectrum contrast is proposed using plasmonic square bracket dimer metasurface. Due to the complex square bracket itself, a dimer structure of two oppositely placed square brackets, and metasurface array configuration, multiple kinds of mode coupling can be devised in the inner and outer elements within the metasurface, enabling four sensing channels with the sensitivities higher than 200 nm/RIU for refractive index sensing. Among them, the special sensing channel based on the reflection-type surface lattice resonance (SLR) mechanism has a full width at half maximum (FWHM) of only 2 nm, a high peak-to-dip signal contrast of 0.82, a high Q-factor of 548, and it can also behave as a good sensing channel for the thickness measurement of the deposition layer. The multi-band sensor can work normally in a large refractive index or thickness range, and the number of resonant channels can be further increased by simply breaking the structural symmetry or changing the polarization angle of incident light. Equipped with unique advantages, the suggested plasmonic metasurface has great potential in sensing, monitoring, filtering, and other applications.

## 1. Introduction

The use of a metal–insulator–metal (MIM) plasmonic metasurface to serve as a narrowband perfect absorber has recently attracted a great deal of attention [1,2,3,4,5]. The metasurface perfect absorber can dissipate the majority of incident light and results in a small quantity of reflection and transmission in the narrowband of the wavelength range. Since the resonant characteristics of narrowband resonances in the MIM plasmonic metasurface are quite sensitive to small changes in its surroundings (e.g., the variation of the ambient refractive index (RI) and adhesion of molecule layers), and such environmental perturbations can be derived by the spectral shifts, this kind of structure has become a considerably ideal candidate to inspect and quantify a small quantity of chemical or biological species in an efficient and nondestructive way. So far, it has been successfully exploited in a large variety of applications, including disease diagnosis [6], liquid refractometer [7], gas detection [8], and biosensing [9,10]. In addition, owing to the extra dependence of the resonance frequency on the structural configuration, such as the element size, shape, material composition, and array arrangement, the wavelength band for applications can be flexibly transferred by adequately designing the metasurface.

Generally, a narrowband resonance in the MIM plasmonic metasurface is tightly correlated to the strong interaction of the metal and electromagnetic fields. For instance, the unique interplay of conductive electrons in the isolated metallic structures and light at a certain frequency can generate the so-called localized surface plasmon resonance (LSPR), providing a classic manner to moderately shrink the resonance linewidth. LSPR additionally exhibits large local field enhancements in deep subwavelength volumes, which is especially attractive for sensing fields [11,12,13]. In addition, the LSPR frequency can be readily governed since it relies on not only the structure design and local environment but also the interaction between surface plasmon modes in the structure simultaneously [14].

However, due to their relatively strong radiative damping, LSPRs usually have a broad linewidth [15], which significantly limits the performance of LSPR-based sensors. The resonance position in the spectrum having a high quality-factor (Q-factor), namely a narrow resonance linewidth that is crucial for optical sensing since a high-Q-factor structure has the ability to measure small resonant wavelength shifts with greater accuracy, imparts the designed devices with good sensitivity (S) and an excellent figure of merit (FOM) value. Actually, sensitivity is an important indicator for optical sensors, and the realization of highly sensitive plasmonic sensors has long been a goal to pursue. For example, Chou et al. proposed a highly sensitive and tunable plasmonic sensor based on a nanoring waveguide resonator with silver nanorods, achieving a sensing sensitivity of 2080 RIU/nm [16]. However, these MIM waveguide plasmonic sensors are not capable of processing spatial signals. Kravets et al. proposed metal–dielectric–graphene hybrid heterostructures with enhanced surface plasmon resonance sensitivity based on amplitude and phase measurements, and the demonstrated maximum sensitivity exceeded 30,000 nm/RIU [17]. Although this structure has amazing sensing performance, it will not be conducive to nanoscale integration due to the use of a prism. For optical sensors, a high peak-to-valley contrast is also essential to improve the signal-to-noise ratio, especially when the detected signal is affected by instrumental noise. In order to improve the performances of optical sensors, substantial efforts have been devoted to increasing the Q-factor of plasmonic sensors, such as utilizing the emerging mechanisms of bound states in the continuum (BICs), surface lattice resonance (SLR), and so on. Unlike traditional bound states, the BIC is completely decoupled from the radiative continuum, though it is located within it [18]. Theoretically, BIC has an infinite Q-factor; however, it cannot be excited directly by the incident wave owing to its decoupling from the radiative channel. In addition, it is not possible for the BIC to be detected because of its zero spectral linewidth. In fact, collapsing a perfect BIC into a quasi-BIC is a feasible way to achieve a high Q-factor that is detectable [19]. The concepts of symmetry-protected BIC and incidental BIC offer some common approaches to engineer metasurfaces with a quasi-BIC. However, traditional designs driven by symmetry-protected BICs and incidental BICs require an extremely small perturbation parameter to obtain very large Q-factors, complicating the sample fabrication process and limiting practical applications [20]. Recently, hyperbolic metamaterials composed of alternating layers of metal and insulator have been considered as another alternative for high-performance sensors [21,22,23]. For instance, Sreekanth et al. proposed an extremely sensitive biosensing platform based on hyperbolic metamaterials; by exciting diverse modes using a grating–coupling technique, they achieved different extreme sensitivity modes with a maximum of 30,000 RIU/nm and a record figure of merit (FOM) of 590 [23]. However, multilayer composite structures have extremely high requirements for processing and fabrication, which to some extent hinder the wide application of hyperbolic metamaterials.

In contrast, SLR-based metasurface sensors require much less in terms of the fabrication process and accuracy, whilst still achieving decent performance. Research shows that if the diffracted light waves propagate exactly in the periodic plane of the array and are coupled with the local resonance of individual nanoparticles when metal nanoparticles are arranged in periodic arrays, i.e., a hybrid mode, the so-called SLR will be formed [24]. In this type of SLR, the radiation loss of every single particle can be suppressed by the diffraction coupling of the array, leading to more significant field enhancement around the array and a corresponding pronounced spectrum feature of a fairly narrow linewidth. Thus, the utilization of the SLR mechanism for high-performance sensing is an ideal choice due to its nature of ultrahigh Q-factor [14]. For example, M. Saad Bin-Alam et al. reported a plasmonic metasurface with a Q-factor of 2340 [25], which has been the highest experimental measurement value so far. Hu et al. proposed dielectric nanocavity-mediated gold nanostructure arrays exhibiting both narrow spectral features with a linewidth of ~8.2 nm and strong resonance intensity with an absorbance amplitude exceeding 95% [26]. Li et al. designed and fabricated a gold nanodisk array-based SLR sensor for the detection of antimouse IgG protein, with a resultant protein sensitivity of up to 1.25 nm/nM [27]. However, a number of studies on SLR responses are on transmission-type spectra, which require an RI matching layer to maintain RI uniformity in the environment around meta-atoms [28,29,30]. The RI matching layer under consideration hinders the direct contact between the meta-atom and the matter under test and limits the practicality and efficiency of sensing [31]. Additionally, most current plasmonic sensors have a single sensing channel [27,32,33], which poses a challenge to improve detection efficiency in limited spaces. In fact, adding a number of resonant modes is an effective way to realize high-performance sensors with a multi-band operating channel.

Here, a four-spectral-band plasmonic optical sensor with distinct resonance dips located in the range of 700–1200 nm is proposed using a MIM square bracket dimer metasurface. By oppositely placing two identical complex square brackets in the metasurface array, the number of resonant modes is increased owing to an improvement in the possibility of mode coupling and hybridization. From the spectra results, all four separate absorption bands show a high absorption ratio and narrow linewidth. More importantly, among these resonances, the reflection-type SLR shows a marvelous full width at half maximum (FWHM) of 2 nm and high peak-to-dip signal contrast of higher than 0.8, and its resonance wavelength can be flexibly modulated by adjusting the period along the y direction. According to the analysis of sensing performance, the proposed sensor can render a high sensitivity of above 200 nm/RIU, and 0.22 nm/nm for the RI and thickness measurement, respectively. Moreover, the Q-factor and FOM of SLR reached 548 and 139 RIU^−1^, respectively. We hope that the proposed metasurface structure can offer great potential in practical sensing applications.

## 2. Structure and Simulation Setup

**Figure 1:**
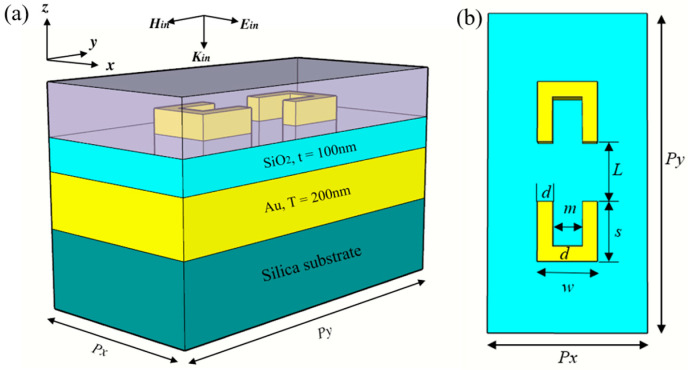
(**a**) Schematic of the plasmonic metasurface sensor that consists of the gold square bracket dimer. (**b**) Top view of the nanostructure. Here, *Px* = 550 nm, *Py* = 1100 nm, *L* = 200 nm, *s* = 200 nm, *w* = 200 nm, *m* = 100 nm, and *d* = 50 nm.

The optimized unit cell has periodic dimensions of *Px* = 550 nm and *Py* = 1100 nm. The heights of the top gold pattern layer, dielectric layer, and slab gold layer are *h* = 75 nm, *t* = 100 nm, and *T* = 200 nm, respectively. The top gold nanostructure parameters are *s* = 200 nm, *d* = 50 nm, *m* = 100 nm, and *L* = 200 nm and are shown in Figure 1b; the medium on the top of the structure is a layer of polymer (*n* = 1.33) with a thickness of 200 nm. The simulated results are based on the finite–difference time–domain (FDTD) numerical method (ANSYS Lumerical Software 2020 R2.4). In our simulations, the periodic structures are illuminated by a normally incident electromagnetic wave with the polarization direction parallel to the x-axis. To obtain accurate and stable results with an acceptable simulation time, the grid sizes are taken as *dx* = 5 nm, *dy* = 5 nm, and *dz* = 5 nm uniformly in x, y, and z coordinates and the simulation time step is chosen as 80,000 fs. Perfectly matched layers (PML) are applied in the z direction and periodic boundary conditions are utilized in the xy plane. The field and charge distributions, reflection spectra, and transmission spectra are obtained by properly setting frequency-domain field and power monitors. The dielectric constants of gold and silica are from the CRC Handbook of Chemistry and Physics and Palik Handbook of Chemistry and Physics, respectively.

## 3. Results and Discussion

### 3.1. Structure Optimization and Feature Analysis

**Figure 2:**
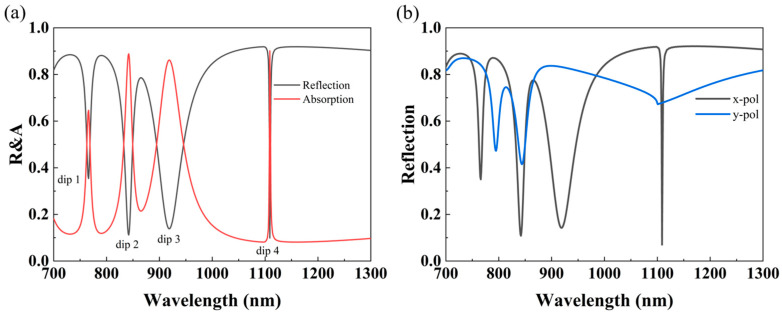
(**a**) The reflection and absorption spectra of the proposed metasurface with the structural parameters *Px* = 550 nm, *Py* = 1100 nm, *h* = 75 nm, *t* = 100 nm, and *T* = 200 nm. (**b**) The reflection spectra excited by orthogonally polarized incidences.

Figure 2a shows the reflection spectra of the square bracket dimer metasurface. There is no transmission through the MIM plasmonic surface, as the gold layer is thick enough to block the incident beam, which will constantly keep the outgoing beam in the same RI space as the incident beam. The absorption A can be computed as A = 1 − R, where R is the reflection. It is clearly seen that there are four reflection dips at around 766 nm, 842 nm, 916 nm, and 1109 nm. For convenience, the four reflection dips are named dip 1, dip 2, dip 3, and dip 4 from low to high wavelengths, respectively. The FWHM of dip 4 is only 2 nm, which shows the largest contrast and high Q-factor compared to the other three dips, and, theoretically, it is more suitable for sensing and monitoring. Due to the unequal length and width of the structure and period, our proposed structure is polarization-dependent, as shown in Figure 2b. It can be found that more modes can be obtained in the case of transverse polarization (parallel to the x-axis), and so we choose a light source with x-polarization.

**Figure 3:**
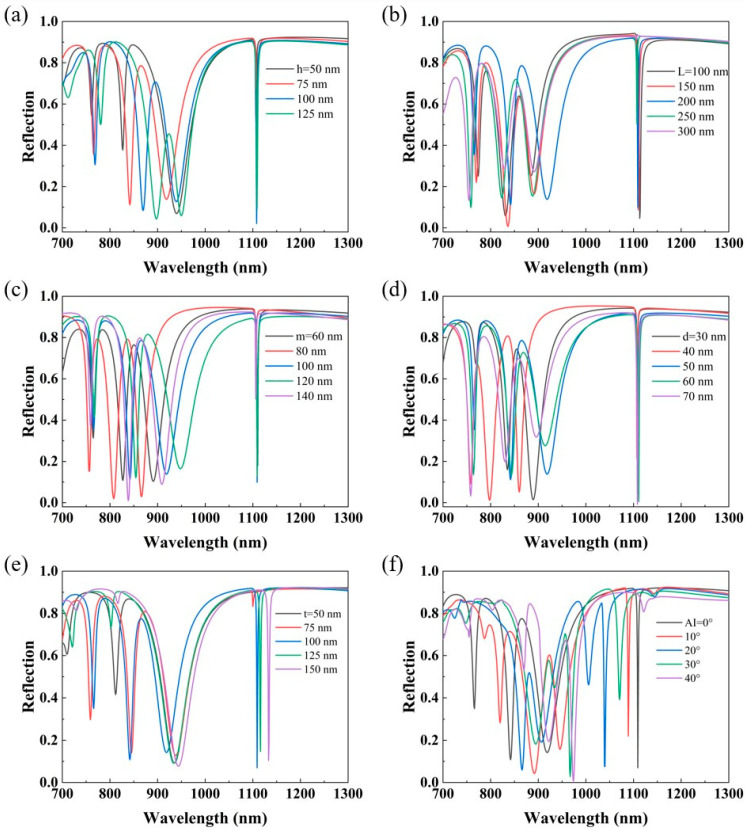
(**a**–**f**) The reflection spectra of the proposed metasurface for different top layer heights *h*, top and bottom bracket spacings *L*, nanobar widths *d*, nanocavity spacings *m*, SiO_2_ layer thickness t, and angle of incidence (*AI*), respectively.

In order to understand the effect of the designed metasurface structural parameters on metasurface performance, we investigated the reflection spectra under the variation of the parameters *h*, *L*, *d*, *m*, *t*, *AI*, *Px,* and *Py* of the gold square bracket dimer. Keeping the other parameters constant, we first calculated the reflection spectra at top layer heights of 50 nm, 75 nm, 100 nm, and 125 nm, as shown in Figure 3a. It can be found that, with the increase in height, dip 1 and dip 2 are red-shifted and the corresponding FWHM gets larger, but the peak-to-dip signal contrast of the latter shows an increasing trend. The FWHM of dip 3 does not change too much, but the peak-to-dip signal contrast is higher when the heights of the gold square bracket dimer are 50 nm and 125 nm. Surprisingly, the resonance position of dip 4 does not notably change in the spectra, but the depth of the resonant spectrum changes a lot. It is worth mentioning that when the height is 100 nm, the absorption of dip 4 almost reaches 99%, and the peak-to-dip signal contrast of dip 4 is greater at a height of 75 nm than that at a height of 50 nm. Combining the above results and considering the stability of the structure fabrication, we finally chose the array height to be 75 nm. Next, we investigated the reflectance spectra for different top and bottom bracket spacings *L* from 100 nm to 300 nm in steps of 50 nm, and the results are shown in Figure 3b. From the results, both dip 1 and dip 4 have blueshifts, and dip 4 has a peak-to-dip signal contrast far higher than those in other cases when *L* = 200 nm. Moreover, the resonance position of dip 4 remains almost unchanged. The variation trends of the resonance positions of dip 2 and dip 3 are similar, but the linewidth of dip 3 shrinks while that of dip 2 increases when *L* gets larger. Then, with the other parameters unchanged, we studied the reflection spectra at different nanobar widths *d* and nanocavity spacing m, respectively, and the results are shown in Figure 3c,d. On the one hand, it can be found that with the increase in nanobar width, the linewidth of dip 4 gradually becomes larger but still remains below 3 nm, and the corresponding peak-to-dip signal contrast of dip 4 also shows an increasing trend. Similarly, we find that the resonance position of dip 4 remains almost unchanged. However, the linewidth of dip 1 decreases as the nanobar width d increases, but its peak-to-dip signal contrast tends to increase. Here, the resonance positions of dip 2 and dip 3 show similar trends, and for both, the highest peak-to-dip signal contrast occurs at *d* = 40 nm, but the narrowest linewidth for dip 2 occurs at *d* = 50 nm and for dip 3 at *d* = 40 nm. On the other hand, it is interesting to note that the resonance positions of all four dips show the same trend as the nanocavity spacing increases, thus showing that the nanocavity spacing has a non-negligible effect on the performance of the metasurface. Overall, the linewidths of the four dips show a decreasing and then increasing trend. Although dip 1, dip 2, and dip 3 all exhibit narrow linewidths and high peak-to-dip signal contrasts at *m* = 80 nm, dip 4 has narrower linewidths and higher peak-to-dip signal contrasts at *m* = 100 nm compared to the values at *m* = 80 nm, and the other three dips also display good performance at *m* = 100 nm. Then, we investigated the reflection spectra for different dielectric layer thicknesses, as shown in Figure 3e. As the thickness of the dielectric layer increases, dip 1 and dip 4 appear red-shifted. At *t* = 100 nm, dip 2 and dip 4 have the narrowest line widths and the highest peak-to-valley signal contrast. While dip 1 has the narrowest linewidth at *t* = 75 nm, dip 4 has a poor linewidth and contrast at this time. In contrast, dip 3 has little change in linewidth and contrast. So, we choose *t* = 100 nm as the thickness of the dielectric layer. The reflection spectra of our proposed structure at different angles of incidence of light source are shown in Figure 3f. It can be found that the relative positions of the four dips change as the angle of incidence increases, where dip 1 and dip 4 appear to be degraded, dip 2 and dip 3 have a tendency to converge, and new resonance peaks appear in the spectra. Therefore, after analyzing the above-calculated results and considering the feasibility of actual processing, we finally confirmed the structural parameters to be *h* = 75 nm, *L* = 200 nm, *d* = 50 nm, *m* = 100 nm, and *t* = 100 nm. It is worth noting that in the actual characterization of the metasurface properties, we have to take into account not only the geometrical parameters of the structure but also the physical, chemical, and other properties of the structural material.

**Figure 4:**
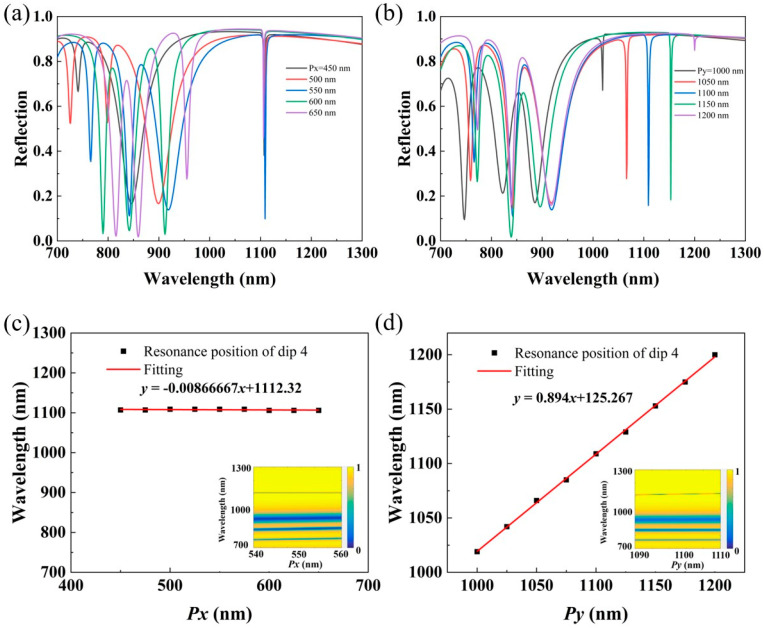
(**a**) The reflection spectra of the proposed metasurface at different *Px* while *Py* = 1100 nm. (**b**) The reflection spectra of the proposed metasurface at different *Py* while *Px* = 550 nm. (**c**) Fitted curve of the resonance position of dip 4 versus *Px*; the inserted chart is the reflection spectrum map of the proposed metasurface with varying *Px*. (**d**) Fitted curve of the resonance position of dip 4 versus *Py*; the inserted chart is the reflection spectrum map of the proposed metasurface with varying *Py*.

**Figure 5:**
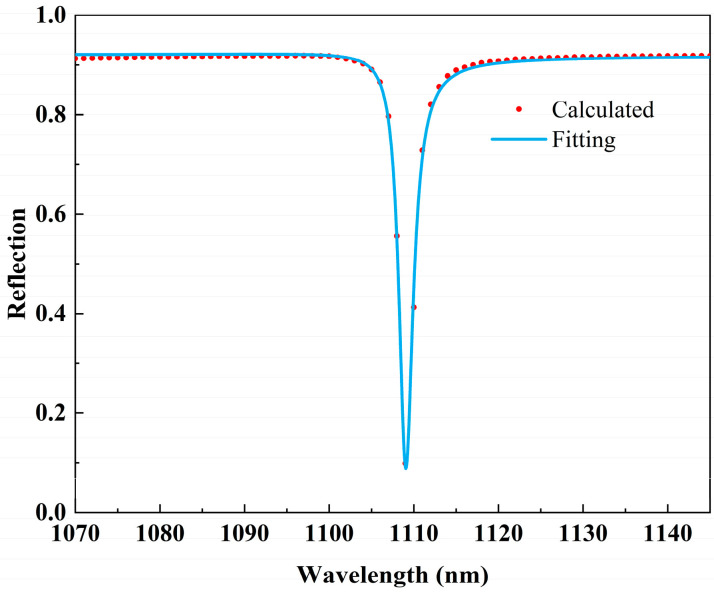
Fano fitting of dip 4 with the structural parameters *Px* = 550 nm, *Py* = 1100 nm, *h* = 75 nm, *t* = 100 nm, and *T* = 200 nm.

Then, we investigated the reflection spectra with respect to different *Px* under the condition of *Py* = 1100 nm, and the results are shown in Figure 4a. It is worth noting that as *Px* increases from 450 nm to 650 nm, the resonance position of dip 4 remains almost unchanged. The peak-to-dip signal contrast is highest at *Px* = 550 nm, and the other three resonance peaks show a red-shift trend as a whole. However, when *Px* = 450 nm, only three resonance peaks appear in the spectrum, which may be due to the loss of resonance modes inside the metasurface that is caused by a reduction in the period. We also studied the cases corresponding to *Py* = 1000 nm, *Py* = 1050 nm, *Py* = 1100 nm, *Py* = 1150 nm, and *Py* = 1200 nm, and the results are shown in Figure 4b. From the results, both dip 1 and dip 4 have red-shifts, and dip 4 has a peak-to-dip signal contrast far higher than those in other cases when *Py* = 1100 nm. The variation trends of the resonance positions of dip 2 and dip 3 are very similar, but the linewidth of dip 2 shrinks whilst that of dip 3 increases when *Py* gets larger. It is worth mentioning that the spectral changes produced by decreasing the spacing *L* between the upper and lower brackets remain almost the same as increasing the period *Py*. In addition, we have fitted the resonance position of dip 4 to *Px* and *Py*, respectively, as shown in Figure 4c,d, and the results show an almost linear relationship between *Py* and the resonance wavelength of dip 4, which establishes a link between the structure and the optical response, and provides theoretical guidance for realizing RI sensors with the target wavelength. Furthermore, the dependence of the reflection spectra of the gold square bracket dimer metasurface under the adjustment of periods *Px* and *Py* are plotted in insets of Figure 4c,d, which further illustrate the linear relationship between *Py* and the resonance wavelength of dip 4. Through the above analysis, we found that changing *Px* within a certain range will not change the resonance position of dip 4, but varying *Py* will cause obvious variations in the resonance position of dip 4 and a visible decrease in contrast. Dip 1 also has a similar trend. We speculate that the formation of dip 1 and dip 4 may be related to the longitudinal spacing of the array. On the contrary, dip 2 and dip 3 are more sensitive to lateral spacing. Furthermore, dip 4 exhibits typical Fano-type resonance features; for the asymmetric linear Fano resonance, the Q factor is calculated as Q = *ω*_0_/2*γ* and fit constants are extracted with the Fano model [34]:

(1) $$T=|a_{1}+ia_{2}+\frac{b}{\omega -\omega_{0}+i\gamma }{|}^{2}$$

where *a*_1_, *a*_2_, and *b* are the fitting constants, *ω*_0_ is the frequency, and *γ* is the overall damping rate. The fitting curves are shown in Figure 5. It is found that the fitted spectrum (the blue solid line) agrees well with the numerically calculated spectrum (the red dots), which further verifies the excitation of Fano-type resonance. With the fitted damping coefficients, the Q-factor of dip 4 at this point can be calculated to be 548. The resonance positions, FWHM, and Q-factors of all four dips are shown in Table 1.

### 3.2. Underlying Mechanisms of Resonant Modes

**Figure 6:**
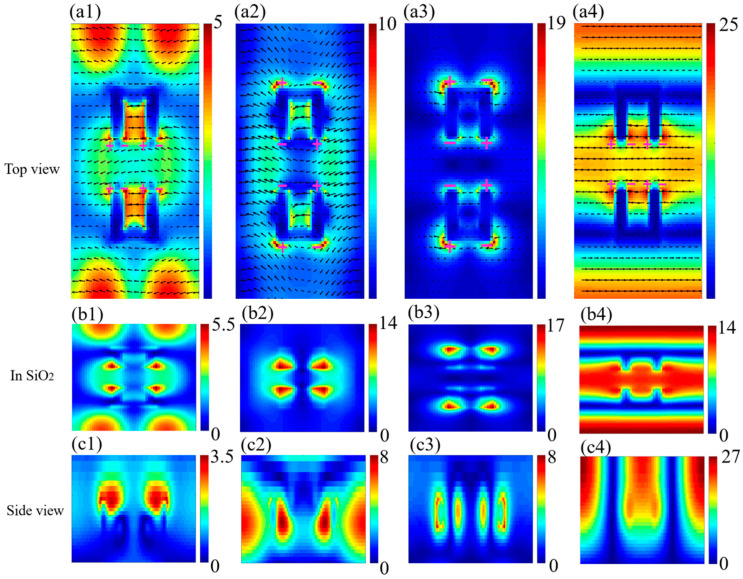
(**a1**–**a4**) Electric field direction (in arrows) and intensity (in color) maps on the xy plane at four resonant dips, dip 1, dip 2, dip 3 and dip 4, respectively; (**b1**–**b4**) and (**c1**–**c4**) show the map in SiO_2_ and side view of E-field distributions at four resonant dips, dip 1, dip 2, dip 3 and dip 4, respectively.

To gain further insight into the underlying physics of the resonance peaks discussed above, the corresponding electric field distributions for the four resonance modes are calculated, as depicted in Figure 6(a1–a4). Each map shows a unit cell of the arrays. The color scale represents the normalized electric field intensity enhancement, while the black arrows denote the real components of the electric field vector projected in the xy plane, and ‘+’ and ‘−’ indicate positive and negative charge distributions, respectively. Figure 6(b1–b4,c1–c4) plots the electric field distributions within the SiO_2_ layer and the side view (yz plane) at four resonance peaks of the spectrum. For dip 2 and dip 3, they have similar electric field distributions that are strongly localized at the four corners of the gold square bracket dimer. From Figure 6(b2,b3), we can find that the electric field of the former is concentrated on the inner cavity of the metal dimers and the electric fields on the inner opposite side are coupled with each other to form a symmetric field distribution on both sides of the structure, which is mainly caused by the unity absorption. However, the electric field of the latter mode is strongly concentrated on the four corners of the outer opposite side, which, combined with the distribution of the electric field in Figure 6(c3), is found to be a typical characteristic of LSPRs that is attributed to the imperfect boundary conditions. For dip 4, the near-field profile shows that most of the electric field is mainly distributed. In the outer edges of the metal dimers and their surroundings, as shown in Figure 6(a4,b4), which suggests the excitation of SLR modes. From Figure 6(a4), it can be seen that the electric field distribution on the metasurface is directional and periodic and is orthogonal to the polarization (x-polarization), and thus the SLR belongs to the (0, ±1) order [31], i.e., the corresponding resonance wavelength and period in the y-direction are positively correlated. This further explains the modulation of the SLR wavelength by the structural parameters, which offers guidance to actualize an RI sensor with a target wavelength. Moreover, this also explains the fact that among the four resonant peaks, this mode has the largest electric field strength and lowest radiative damping. At the same time, it also confirms our previous conjecture. Unlike pure LSPR, where the field intensity is mainly confined to the surface of the nanostructures, the pattern of electric field intensity that is observed in the SLR mode extends to the crystal cells [35,36]. For dip 1, we find that its electric field distribution is very special, as shown in Figure 6(a1); part of it is concentrated on the inner cavity of the metal nanostructures, which is similar to that of dip 2. The other part is concentrated on the distal end of the four corners of the outer side of the metal dimers that is most likely caused by a coupling effect formed between the adjacent dimers in the y-axis direction, considering the periodicity of the arrays. Both the internal coupling within the unit cell and external coupling between the adjacent unit cells simultaneously contribute to the substantial reduction in radiation loss of the metasurface, resulting in a narrower linewidth for dip 1 compared to dip 2 and dip 3, but the intensity is much lower when compared to that of the SLR.

### 3.3. Potential Application and Performance

**Figure 7:**
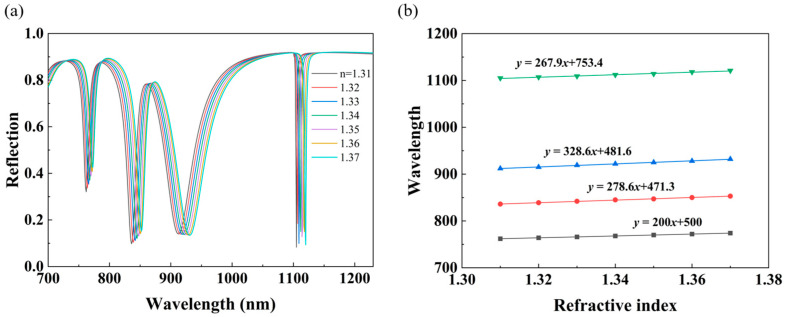
(**a**) Reflection spectra of different refractive indices with the structural parameters *Px* = 550 nm, *Py* = 1100 nm, *h* = 75 nm, *t* = 100 nm, and *T* = 200 nm. (**b**) The dependence of the resonance wavelengths of four dips (from bottom to top: dip 1, dip 2, dip 3, and dip 4) on the refractive index of the cladding.

**Figure 8:**
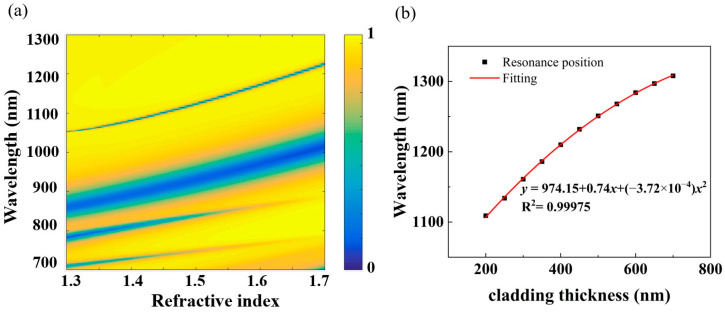
(**a**) Reflection spectrum map from a refractive index of 1.3 to 1.7 for multi-band sensors (from bottom to top: dip 1, dip 2, dip 3, and dip 4). (**b**) Fitting curve of the cladding thickness and the resonance position of dip 4.

The proposed metasurface optical sensors are characterized by multiple channels, a narrow linewidth and high contrast, and can be used for refractive index sensing in addition to filtering and narrowband absorption. So, to verify the sensing effect of the proposed metasurface, we investigated the sensitivity, Q-factor, and FOM of the structure with optimized parameters. Usually, sensitivity (S) and FOM are the idiomatic criteria to characterize the refractive index sensing performance, which are expressed by the following formulas [37]:

(2) $$S=\frac{\Delta \lambda }{\Delta n},$$

(3) $$FOM=\frac{S}{FWHM}$$

where ∆λ is the resonance wavelength shift caused by the ambient refractive index change and *FWHM* is the full width at half maximum of the mode. We set refractive indices of the polymer cladding above the metasurface from 1.31 to 1.37 in steps of 0.01, and the corresponding reflection spectra are shown in Figure 7a, while the fitting curves between the resonant wavelength and the refractive index of cladding are displayed in Figure 7b. We can see that there is a significant red-shift in the resonance position of the structure as the refractive index of cladding increases. From the fitting curves, the resonant wavelength and refractive index show a good linear relationship and the corresponding sensitivity (S) of the four dips (from bottom to top) can be obtained, which are around 200 nm/RIU, 278.6 nm/RIU, 328.6 nm/RIU, and 267.9 nm/RIU, respectively. From Table 1, we can see that the corresponding FWHMs of the four dips from left to right are 9.5 nm, 38.1 nm, 45.8 nm, and 2 nm, respectively. The calculated Q-factors are 80.6, 22.1, 20.1, and 548 for the four dips, and the FOM of dip 4 reaches 134 RIU^−1^ due to the ultra-narrow linewidth. The goodness of fit R^2^, root mean square error (RMSE), and refractive index (RI) error for each resonance peak are shown in Table 2, and it can be found that our metasurface sensor has good accuracy. The spectral resolution of one spectrometer to sense sensitivity decides the RI resolution in the system. If a resolution of 0.05 nm for the spectrometer is assumed, the theoretical limit of our metasurface sensor will be 2.5 × 10^−4^ RIU. Furthermore, in order to illustrate the advantages of our nanostructure, comparisons of FWHM, S, FOM, and the quantity of sensing channels between this work and some recently reported structures are described in Table 3. Among the four sensing channels, even though dip 4 possesses the highest Q-factor, its sensitivity is lower than that of dip 2 and dip 3, which is obviously not in line with our expectations. This phenomenon can be explained as follows: in fact, there is a direct relationship between the Q-factor and sensitivity pointed out by Li et al. in a proposed SLR-based plasmonic sensors [27]. Namely, the sensitivity of optical sensors is related to the near field. When the near field is strongly localized, the far-field radiation loss controlled by the Fourier dual of the field distribution is high. As a result, the Q-factor of the resonator is low. In our proposed structure, dip 4 originates from SLR, and although SLR greatly suppresses the radiation loss in the far field, the corresponding localization strength in the near field is much weaker compared to dip 3, and so the sensitivity of dip 4 is lower than that of dip 3. In addition, as shown in Figure 8a, the proposed multi-band sensor works normally in the refractive index range from 1.3 to 1.7, which greatly helps to broaden the application scenarios of the sensor.

Furthermore, the relationship between the cladding thickness and wavelength position of SLR is investigated as well. From the results and fitting line in Figure 8b, one can see that there is a certain correlation between these two variables. In other words, the thickness variation of the cladding can be accurately derived from the wavelength shift of SLR. Thus, this metasurface-based platform can also be applied to the thickness measurement of the cladding, providing a simple method to evaluate the deposition quality or to obtain the effect of a dose test in nanofabrication. In terms of its performance, it is seen that the sensitivity is higher (e.g., ~0.6 nm/nm at a thickness of 200 nm) when the thickness of the cladding layer is relatively low because the slope of the fitting line gradually decreases with the increase in thickness. Fortunately, in this large thickness span of 200–700 nm, the sensitivity exceeds 0.22 nm/nm, which is workable in real conditions as the wavelength shift can be easily detected by most commercial spectrometers even when the layer thickness varies by one nanometer.

The proposed metasurface definitely manifests improved sensing performance with multiple sensing channels, which expands the sensing bands and indicates its great potential in the field of sensing. Especially, SLR (dip 4) is expected to work in biochemical sensing and thickness measurement, benefiting from the excellent performance indicators of a narrow FWHM, high Q-factor, and high sensitivity.

### 3.4. Realization of More Resonant Modes

**Figure 9:**
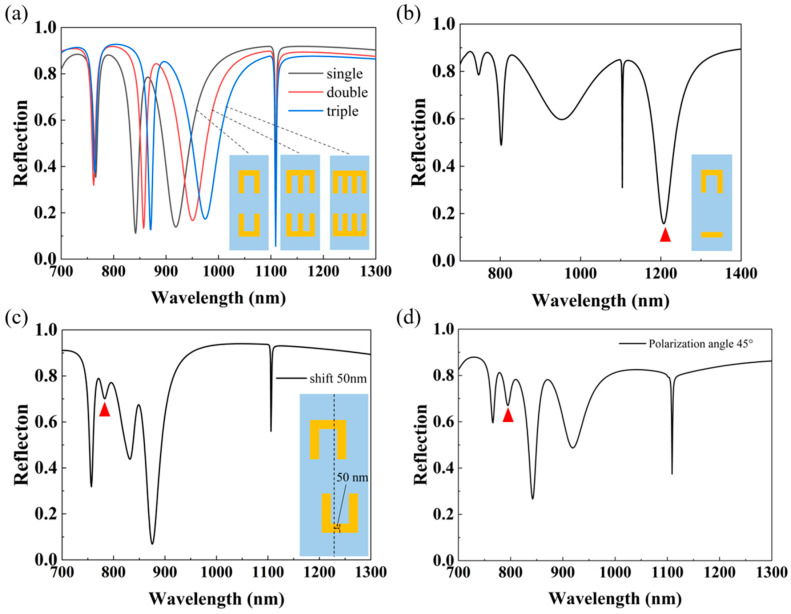
(**a**) Reflection spectra of multi-band sensors with different numbers of brackets. The width d of the nanobars is 50 nm, and the spacing of the longitudinal nanobars is 100 nm, 75 nm, and 50 nm from left to right, respectively. (**b**) Reflection spectra of muti-band sensors with a symmetry-breaking structure. (**c**) Reflection spectra of muti-band sensors with a shift of 50 nm of the upper and lower brackets. (**d**) Reflection spectra of muti-band sensors when the polarization angle of incident light is set to 45°. The positions of the new resonance dips are marked by red triangles.

More resonant modes in the metasurface distinctly correspond to more useful sensing or filtering channels, which is instructive to some specific applications, such as optical multi-switch [41], multi-band absorption [42]. While keeping the structural period constant, we rationalize the structural layout to investigate the reflection spectra of single-bracket-, double-bracket- and triple-bracket-based metasurfaces, respectively. The shapes of the unit cells and the calculated results are depicted in Figure 9a. Surprisingly, with the increase in the number of brackets, the number of resonance dips is constant and the resonance positions of dip 1 and dip 4 are almost unchanged, but dip 2 and dip 3 are red-shifted at the same time. This can be explained by the following reasons: when the number of brackets is increased from one to three, the coupling within the cavity of a single bracket is enhanced at the same time as the coupling between the upper and lower brackets is enhanced due to the constant period. This local field enhancement is what causes the red-shift of the spectral lines, and a similar trend can be compared to Figure 3c,d. This phenomenon further verifies the linear relationship between the resonance wavelength of the SLR and the period *Py*, indicating that the suggested structure not only has a small footprint but also a large fabrication tolerance. Here, we can also find that since the structural symmetries along the x- and y-axes still hold, merely increasing the number of the connected brackets cannot generate extra resonances.

In addition to the way to increase the resonant modes by changing the angle of incidence of the light source mentioned in Figure 3f, we have further investigated other ways as well. First, we broke the symmetry of the whole structure by reducing the arm length of the lower bracket so that it became a nanobar, as shown in Figure 9b. From the reflection spectrum, a new resonance dip (labeled by a red triangle) located on the right side of the SLR successfully appears. Similarly, another symmetry-breaking manner in which the upper bracket is left shifted while the lower bracket is right shifted with the same distance is also considered. The related reflection spectrum is shown in Figure 9c with an exemplary shift distance of 50 nm. Apparently, a new resonance marked by a red triangle appears between the initial dip 1 and dip 2. Thus, properly breaking the structural symmetry can efficiently actualize an increase in the number of resonances. On top of that, the location of new resonances could be different, depending on the different configurations of asymmetric structures, as in the two metasurfaces illustrated in Figure 9b,c.

Given that the reflection behavior of the considered metasurface is sensitive to incident polarization, another feasible approach to produce more resonances is to change the polarization angle of the incident light. As shown in Figure 9d, when the polarization angle of the incident light is set to 45°, a new resonance dip occurs between dip 1 and dip 2, which further enriches the means of realizing multi-band sensors.

### 3.5. Manufacturing Procedure of the Metasurface

**Figure 10:**
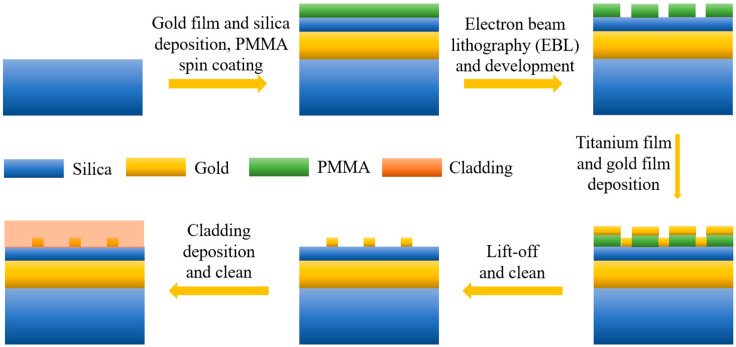
Manufacturing procedure chart of silica-based MIM metasurface.

As to the fabrication of the proposed plasmonic metasurface, a possible process is listed as follows. The surface of the silica substrate will be first cleaned. Then, a 200 nm-thick gold film will be deposited on the substrate, followed by successively depositing 100 nm-thick silicon dioxide and spinning 100 nm-thick polymethylmethacrylate (PMMA) photoresist layers on the gold film. Then, an array of gold square bracket dimers will be identified using electron beam lithography (EBL) and this pattern will be transferred to PMMA by developing. Next, a 5 nm-thick titanium layer and a 75 nm-thick gold layer will be deposited in sequence using electron beam evaporation to enhance adhesion. Subsequently, the lift-off and cleaning procedures will result in the desired gold nanoparticle arrays. Finally, a cladding with a thickness of 200 nm will be deposited and the surface layer will be subsequently cleaned. The manufacturing procedure chart of the MIM metasurface is shown in Figure 10.

## 4. Conclusions

In conclusion, a multi-band plasmonic metasurface with a narrow linewidth, high Q-factor, and high peak-to-dip signal contrast is proposed, benefitting from the diverse modes of coupling and hybridization. Such a metasurface can serve as a sensor to measure the refractive index and thickness of the deposition layer, with high sensitivities exceeding 200 nm/RIU and 0.22 nm/nm, respectively. Additionally, the SLR sensing channel can be manipulated by modifying the parameter of period length along the y direction, offering a feasible and easy route to transfer the sensing band. The number of sensing channels can be extended efficiently by the structural asymmetry and variation of the incident polarization angle. The proposed optical sensor can work properly with a large refractive index and thickness range, expanding the workable scenarios efficiently. Owing to the small size, simple structure, large working range, and good performance, the proposed metasurface exhibits good prospects for applications in biosensing and monitoring.

## Figures and Tables

**Table 1:** The resonance positions, FWHM, and Q-factors of all four dips.

Results	Dip 1	Dip 2	Dip 3	Dip 4
Resonance wavelength (nm)	766	842	916	1109
FWHM (nm)	9.5	38.1	45.8	2
Q-factor	80.6	22.1	20.1	548

**Table 2:** Goodness of fit, root mean square error, and refractive index error for each resonance peak.

Results	Dip 1	Dip 2	Dip 3	Dip 4
R^2^	1	0.99803	0.99811	0.98754
RMSE (nm)	-	0.2928	0.3380	0.7121
RI error (RIU)	-	1.05 × 10^−3^	1.03 × 10^−3^	2.66 × 10^−3^

**Table 3:** Comparisons of optical properties between some recently proposed nanostructures.

Reference	FWHM (nm)	S (nm RIU^−1^)	Q-Factor	Quantity of Sensing Channel
[38]	2.01	1661	728	2
[39]	5.8	693.9	-	2
[32]	295	330	4.52	1
[33]	6.0	474.7	162	1
[40]	2.0	161.5	599	2
This work	2.0	267.9	548	4

## Data Availability

The data presented in this study are available on request from the corresponding author.
